# Supplementary material for: The Impact of Qualification and Hospice Education on Staff Attitudes during Palliative Care in Pediatric Oncology Wards—A National Survey
Source: Children (Basel). 2024 Feb 1;11(2):178. doi: 10.3390/children11020178 (PMC10886866; doi:10.3390/children11020178)
Supplement: Supplementary file 1 [file children-11-00178-s001.zip › children-2757960-supplementary.pdf]

Supplementary Materials

Table S1. Interview

Topic	Question
INTRODUCTION OF PALLIATIVE THERAPY, FIRST COMMUNICATION	1. Will palliative care be introduced at some point in your department for patients who cannot be cured?<br><br>2. When palliative care be introduced in your department?<br><br>3. If you have had an example of palliative care being introduced at the time of initial diagnosis, have you seen any benefits or disadvantages of this in subsequent care?<br><br>4. How is the first communication made? Who is present from the family and staff?<br><br>5. Does the doctor talk to the parents alone, or is the first communication done in a team? Which do you prefer and why (at least three reasons)?<br><br>6. Why might the child's presence at the first communication be justified? Conversely, why might the child's absence be justified?<br><br>7. Talking to the child about the therapy/consequences/outcome, how does it affect the process from the staff's perspective?
SYSTEM OF PALLIATIVE CARE	8. What criteria would you use to decide between home-based and hospital-based palliative care for a patient? Why?<br><br>9. How does the family's attitude towards the place of care influence this decision?<br><br>10. Does the depth of your relationship with a particular child influence your decision about hospital or home palliative care? If you have a more personal relationship with the patient, which care do you prefer?<br><br>**11. What is the three most common difficulties in order of frequency in implementing palliative care?**<br><br>12. What organizational steps could be taken to influence this difficulties encountered in the care process?<br><br>13. What do you think are the advantages and disadvantages of home-based hospice care?<br><br>14. What do you think are the advantages and disadvantages of hospital-based hospice care?<br><br>15. Which is more physically/mentally demanding for you? Why?<br><br>16. Do you use an objective scale, or do you make subjective judgments when assessing patients' complaints (pain, itching, nausea, vomiting, etc.) in palliative care? What do you think of the method you use?<br><br>17. Who do you think are the most important medical providers, from the staff, in palliative care (in order of importance)?

	18. Do you think there is one essential provider from the staff in palliative care?<br>19. What is the ideal frequency of contact with the patient's family in palliative care if it is not in the hospital (daily/weekly/monthly)? Why?
THE TERMINAL STATE	**20. Are the symptoms of the terminal condition (turning over day and night, skin symptoms, irregular and loud breathing, etc.) discussed whit the family?**<br>21. When do you think is the best time to talk about the symptoms of a terminal state?<br>22. Could the discussion about the terminal state be early with adverse consequences for the patient, the environment or care?<br>**23. Could the discussion about the terminal state be late with adverse consequences for the patient, the environment or care?**<br>24. What do you think is the most important issue about dying or death for parents?<br>25. What do you think is the most important issue about dying or death for the child?<br>**26. What is the most important issue about the dying or death of a child from your perspective?**<br>**27. A question about your experience of losing a child, or more specifically, the journey leading up to it: Have there been times when it was a success, have there been times when it was a failure? Give an example of each!**<br>28. If a child cared for in the ward dies, and you have not met the parents, do you find it necessary to contact them? If so, when do you consider it ideal to contact the parents?<br>29. If you do contact the parents by telephone (or other means) at some point after the child's death, why do you do so?
SUMMARY	**30. When do you consider a child's palliative care appropriate?**<br>**31. When do you consider a child's palliative care inappropriate?**<br>**32. If you had the opportunity to change three things about how your ward provides palliative care, what would those three things be? Give reasons why!**<br>33. What are the things that have made a positive impression on you aboute palliative care so far, and why?

*Questions, used in content alaysis are marked in bold.

Table S2. Sample Characteristic – by centers

Total 73 interviews						
**Centers**	**Total Number**	**Age**	**Years in ward**	**Gender (female-male)**	**Qualification (doctor-nurse)**	**Hospice participation (yes-**

						**no)**
**I.**	12	38.92 ± 10.47	9.13 ± 5.53	91.7 – 8.3 %	25 – 75 %	58.3 – 41.7 %
**II.**	14	37.14 ± 10.37	9.21 ± 10.86	78.6 – 21.4 %	57.1 – 42.9 %	14.3 – 85.7 %
**III.**	11	38.10 ± 10.82	13.01 ± 11.86	90.9 – 9.1 %	45.5 – 54.5 %	0 – 100 %
**IV.**	11	39.82 ± 9.52	11.00 ± 9.45	81.8 – 18.2 %	45.5 – 54.5 %	18.2 – 81.8 %
**V.**	9	45.0 ± 7.02	19.33 ± 13.30	100 – 0 %	22.2 – 77.8 %	0 – 100%
**VI.**	6	35.33 ± 11.36	8.68 ± 10.93	66.7 – 33.3 %	50 – 50 %	0 – 100%
**VII.**	10	44.00 ± 10.07	17.60 ± 13.47	100 – 0 %	40 – 60 %	10 – 90 %
**"p"-value**	-	p=0.35	p=0.184	p=0.35	p= 0.628	**p= 0.003**

Table S3. Results of narrative analysis, complete data set

Grouping	Result Median (IQR)	"p"-value
Whole cohort	active verb 2.32 (0.94) > passive verb 0.53 (0.29)	p < 0.001
	constraint 0.58 (0.32) > intention 0.37 80.27)	p < 0.001
	total emotion 1.04 (0.37) > total evaluation 0.93 (80.35)	p= 0.025
	evaluation: positive 0.69 (0.31) > negative 0.21 (0.14)	p < 0.003
	emotion: negative 0.58 (0.32) > positive 0.45 (0.26)	p = 0.005
	positive evaluation 0.69 (0.31) > positive emotion 0.45 (0.26)	p < 0.001
	negative emotion 0.58 (0.32) > negative evaluation 0.21 (0.14)	p < 0.001
	experiential form 16.17 (2.82) > metanarrative and retrospective form 3.43 (2.04)	p < 0.001
Correlations	constraint and Self-reference, weak negative correlation R = -0.247	p=0.035
	positive emotion and We-reference, weak positive correlation R = 0.255	p = 0.029
**Dependent**	Active verb: Nurse 2.36 (1.03) > Doctor 2.18 (0.98)	p=0,360

**on qualification**	Passive verb: Doctor 0.51 (0.22) > Nurse 0.53 (0.33)	p=0,827
	Constraint: Doctor 0.63 (0.34) > Nurse 0.58 (0.35)	p=0,686
	Intention: Doctor 0.39 (0.20) > Nurse 0.36 (0.35)	p=0,439
	**Self-reference: Nurse 3.32 (2.07) >Doctor 2.89 (1.76)**	**p=0,019**
	We-reference: Doctor 2.76 (0.81) > Nurse 2.56 (4.79)	p=0,973
	Psychological perspective: Nurse 2.39 (1.05) > Doctor 2.23 (0.82)	p=0,262
	Cognition: Nurse 1.28 (0.67) > Doctor 1.21 (0.57)	p=0,797
	Emotion (all): Nurse 1.08 (0.38) > Doctor 0.95 (0.31)	p=0,099
	Emotion positive: Nurse 0.48 (0.28) > Doctor 0.41 (0.28)	p=0,119
	Emotion negative: Nurse 0.59 (0.38) > Doctor 0.56 (0.28)	p=0,508
	Evaulation (all): Nurse 0.95 (0.38) > Doctor 0.85 (0.27)	p=0,186
	Evaulation positive: Nurse 0.71 (0.36) > Doctor 0.66 (0.28)	p=0,073
	Evaulation negative Doctor 0.22 (0.14)> Nurse 0.19 (0.14)	p=0,560
	Experiential perspective form: Nurse 16.32 (2.08) > Doctor 15.29 (3.30)	p=0,125
	**Metanarrative perspective form: Nurse 3.64 (2.42) >Doctor 3.14 (1.71)**	**p=0,041**
	Retrospective perspective form: Nurse 3.67 (1.08) > Doctor 3.19 (1.00)	p=0,072
	**Negation: Nurse 4.44 (1.63) > Doctor 3.69 (1.29)**	**p<0,001**
**Dependent on hospice education**	Active verb: Provider 2.45 (0.99) > Non provider 2.32 (0.99)	p=0,405
	**Passive verb: Provider 0.68 (0.56) > Non provider 0.50 (0.27)**	**p=0,022**
	Constraint: Provider 0.70 (0.30) > Non provider 0.58 (0.34)	p=0,582
	Intention: Provider 0.41 (0.26) > Non provider 0.36 (0.27)	p=0,552
	**Self-reference: Non provider 3.23 81.67) > Provider 2.05 (3.01)**	**p=0,033**
	We-reference: Provider 2.76 (0.91) > Non provider 2.61 (0.89)	p=0,645
	Psychological perspective: Provider 2.81 (0.99)> Non provider 2.27 (0.89)	p=0,190
	Cognition: Provider 1.35 (0.57) > Non provider 1.19 (0.62)	p=0,422
	Emotion (all): Provider 1.16 (0.43) > Non provider 1.02 (0.41)	p=0,222
	Emotion positive: Provider 0.48 (0.18) > Non provider 0.44 (0.29)	p=0,284
	Emotion negative: Provider 0.62 (0.43) > Non provider0.59 (0.36)	p=0,613
	Evaulation (all): Non provider 0.94 (0.34) > Provider 0.90 (0.55)	p=0,755
	Evaulation positive: Provider 0.71 (0.34) > Non provider 0.68 (0.33)	p=0,562
	Evaulation negative: Provider 0.21 (0.16) > Non provider 0.19 (0.13)	p=0,304
	Experiential perspective form: Non provider 3.43 (1.00) > Provider 3.14 (1.16)	p=0,311
	Metanarrative perspecitve form: Non provider 3.50(2.24) > Provider 2.81(1.80)	p=0,176
	Retrospective perspective form: Non provider 3.04(0.89) > Provider 2.75(1.10)	p=0,841
	Negation: Provider 4.03(1.90) > Non provider 3.94(1.60)	p=0,823

* From the numbers obtained by Narrkat, we created ratios concerning the total number of words. These ratios were used and compared by Mann-Whitney U-test. The results of the comparisons are given as median (IQR) values. Significant results are marked in bold.

Table S4. Results of the thematic analysis – whole cohort

Question	Response	Result
What is the three most common difficulties in order of frequency in implementing palliative care?	lack of infrastructure	28%
	lack of definition	34%
	psychological burden	73%
	pain relief	24%
If you had the opportunity to change three things about how your ward provides palliative care, what would those three things be?	developing infrastructure	67%
	increasing psychological support	34%
	earlier introduction of palliation	16%
	developing protocols, definitions	26%
How do you experience the loss of a child, or more specifically, the journey leading up to it? Have there been times when it was a success, have there been times when it was a failure?	it can be a success	64%
	it cannot be a success	16%
	Undecided	19%
Are the symptoms of the terminal condition discussed whit the family?	Yes	72,6%
	No	17,8%
	Undecided	9,6%
Could the discussion about the terminal state be late? Does this have any damaging consequences for the patient, environment, or care?	Yes	86%
	No	7%
	Undecided	7%
When do you consider a child's palliative care appropriate?	support of the child needs	65,7%
	preparation of the parents, communication	50,7%
	timely introduction of palliation	12,3%
	team/ professional aspects	19,1%
	Undecided	8,2%
When do you consider a child's palliative care inappropriate?	lack of needs of the child	45,2%
	lack of communication	46,5%
	the belated introduction of palliative care	15%
	team/ professional aspects	17,8%
	Undecided	5,5%
	there is no such thing as inappropriate	6,8%
What is the most important issue about the dying or death of a child from your perspective?	support of the parents needs	23,3%
	support of the child needs	61,6%
	team/ professional aspects	12,3%
	clear communication	10,9%

	Loss/ Grief	17,8%

** For question groups 2, 3, 7, 8, and 9, the % value indicates the percentage of people who answered "yes" to the aspect. For individual responses, yes/no gives 100%.
